# Clinical Assessment of Functional Recovery Following Nerve Transfer for Traumatic Brachial Plexus Injuries

**DOI:** 10.3390/ijerph191912416

**Published:** 2022-09-29

**Authors:** Yi-Jung Tsai, Chih-Kun Hsiao, Fong-Chin Su, Yuan-Kun Tu

**Affiliations:** 1 Department of Medical Research, E-Da Hospital, Kaohsiung 82445, Taiwan; 2 Medical College, I-Shou University, Kaohsiung 82445, Taiwan; 3 Department of Biomedical Engineering, National Cheng Kung University, Tainan 70101, Taiwan; 4 Medical Device Innovation Center, National Cheng Kung University, Tainan 70101, Taiwan; 5 Department of Orthopedics, E-Da Hospital, Kaohsiung 82445, Taiwan

**Keywords:** nerve transfer, rehabilitation, injury levels

## Abstract

Surgical reconstruction and postoperative rehabilitation are both important for restoring function in patients with traumatic brachial plexus injuries (BPIs). The current study aimed to understand variations in recovery progression among patients with different injury levels after receiving the nerve transfer methods. A total of 26 patients with BPIs participated in a rehabilitation training program over 6 months after nerve reconstruction. The differences between the first and second evaluations and between C5–C6 and C5–C7 BPIs were compared. Results showed significant improvements in elbow flexion range (*p* = 0.001), British Medical Research Council’s score of shoulder flexion (*p* = 0.046), shoulder abduction (*p* = 0.013), shoulder external rotation (*p* = 0.020), quantitative muscle strength, and grip strength at the second evaluation for both groups. C5–C6 BPIs patients showed a larger shoulder flexion range (*p* = 0.022) and greater strength of the shoulder rotator (*p* = 0.004), elbow flexor (*p* = 0.028), elbow extensor (*p* = 0.041), wrist extensor (*p* = 0.001), and grip force (*p* = 0.045) than C5–C7 BPIs patients at the second evaluation. Our results indicated different improvements among patients according to injury levels, with quantitative values assisting in establishing goals for interventions.

## 1. Introduction

Traumatic brachial plexus injuries (BPIs) always lead to functional impairment in the upper extremity, for which surgery has remained the treatment of choice for restoring motor and sensory function [1,2,3,4,5]. Various reconstructive procedures have been reported over the recent decades, the functional outcomes of which have been acceptable in traumatic BPI patients with C5–C6 or C5–C7 injuries [2]. Paralysis of the brachialis, biceps, deltoid, and rotator cuff has been found in patients with C5–C6 injury, with additional paralysis of the triceps and wrist extensors having been shown in patients with C5–C7 injury. To allow sufficient upper extremity function during daily activities, restoring shoulder elevation, abduction, external rotation, and elbow flexion should be the primary goals of treatments.

Nerve transfer has remained one of the most optimal treatments for BPI patients, and associated knowledge regarding surgical techniques has been reported. For patients with C5–C6 or C5–C7 injuries, several intraplexus (e.g., median nerve, ulnar nerve, and radial nerve) and extraplexus (e.g., spinal accessory nerve, intercostal nerve, and phrenic nerve) have been considered as the donor nerves. The reinnervation of the suprascapular nerve (SSN) and axillary nerve (AN) to recover the shoulder function has often been set as the primary goal of surgical restoration. The SSN innervates the supraspinatus and infraspinatus muscles, whereas the AN innervates the deltoid and teres minor muscles. Both nerves are responsible for shoulder elevation, external rotation, and abduction. The spinal accessory nerve (SAN) transfer to the SSN can be performed via the anterior supraclavicular approach or posterior approach. These procedures have been found to have satisfactory outcomes, with success rates of achieving shoulder function reaching approximately 80% [6]. Transferring the triceps branch of the radial nerve (RN) to the AN has been reported with good results [7]. The combination of procedures for transferring the SAN to SSN and RN to AN has commonly been used to recover shoulder function in BPI patients with C5–C6 or C5–C7 injuries.

The Oberlin method is a technique commonly used to restore elbow flexion function nowadays. Oberlin et al. described the procedure as the transfer of the ulnar nerve fascicle to the biceps motor branch of the musculocutaneous nerve in order to restore elbow flexion function [8]. Moreover, the combination of the transferring median and ulnar nerve fascicles to the brachialis and biceps has been reported to increase elbow flexion function [1,9]. Few cases of ulnar nerve deficit or decreased grip strength have been reported after the Oberlin procedure, and most patients with C5–C6 or C5–C7 injuries achieved elbow flexion function scores better than M3 [8,10,11,12,13,14].

In addition to the proper reconstruction procedures, adequate postoperative rehabilitation programs are also crucial for improving functional outcomes [9,15,16]. Novak stated that protecting and maintaining the range of motion following the immobilization period are rehabilitation goals for patients in the early phase after nerve transferring; whereas, motor re-education and muscle balance restoration should be emphasized in the late phase [16]. For patients undergoing nerve reconstruction, it is necessary to learn how to recruit the reinnervated muscles by initiating the contraction of the muscles supplied by the donor nerve. The re-education technique depends on the type of nerve transfer procedure. After the Oberlin procedure, patients are often asked to flex their fingers and wrist before performing elbow flexion. These patients need to perform a grip at the initial movement in order to assist with elbow flexion function. Liverneaux et al., who evaluated BPI patients with upper arm involvement receiving the Oberlin II procedure with a training protocol of grip strength and active supination for over 9 months, showed that all patients could achieve an elbow flexion score of M4, with a mean lifting weight of 3.7 kg [9].

The postoperative rehabilitation interventions are important, and outcomes should include results combining the postoperative recovery and rehabilitation interventions for those BPI patients. In addition, diversity might be found in patients with different injury levels even among those who underwent similar reconstruction methods or rehabilitation interventions. Understanding the progress of recovery can be beneficial for setting appropriate treatment goals in actual clinical practice. Therefore, we would like to investigate the functional outcomes of BPI patients following reconstruction of nerve transfer and compare the improvements in those patients with varying injury levels. We focused on recovery progression in patients with C5–C6 and C5–C7 BPIs. The null hypothesis was that post-intervention improvements would be similar in both groups.

## 2. Patients and Methods

Patients with C5–C6 adult traumatic brachial plexus injuries with or without C7 lesions who underwent surgical reconstruction for shoulder and elbow function were recruited in the current study. Injury levels were confirmed via electromyography, nerve conduction velocity data, physical examination, and intraoperative electrodiagnostic data. The inclusion criteria were: (1) patients that underwent reconstruction with neurotization of the spinal accessory nerve (SAN) on the suprascapular nerve (SSN) and Oberlin’s method for functional recovery of the shoulder and elbow by an experienced surgeon, and (2) patients that could follow the instructions and perform the exercise at home. Patients that presented with severe pain, joint contracture in the upper extremities, other neuromusculoskeletal problems (e.g., clavicle fracture, traumatic brain injury, stroke), obstetric brachial plexus palsy, or other reconstructions that might affect assessment were excluded. Patients’ epidemiological data, injury level, trauma mechanisms, and injury side were recorded upon initial evaluation. The study was approved by the Institutional Review Board, and written informed consent was obtained from all subjects before evaluation.

The range of motion, British Medical Research Council (BMRC) scores, quantitative muscle strength, and grip force was measured as the functional outcomes. The range of motion (ROM) during shoulder flexion, abduction, external rotation, elbow flexion, and wrist extension were measured using the goniometer. BMRC scores were measured with the patient in a seated position [17]. The patients were required to keep their trunks as erect as possible during the examination, and the examiner would help stabilize the proximal region to prevent compensatory movement and provide support. Patients who could not perform active movement against gravity (<M3) were defined as having poor outcomes. Quantitative muscle strength was measured using a handheld dynamometer (MicroFET2; Hoggan Health Industries Inc., Salt Lake City, UT, USA) by a well-trained physiotherapist. The measurement range of the dynamometer is 0.8–300 lb, with an accuracy of 0.1 lb. All dynamometer measurements were conducted in the supine position for better stability of the trunk and scapular regions [18]. The patients underwent one or two practice trials with submaximal force to ensure that they understood how to push against the dynamometer and generate force correctly. The maximum isometric strength of each muscle group was then evaluated, with the non-affected arm being tested first. The muscle groups of shoulder flexor/abductor/external rotator, elbow flexor/extensor, and wrist flexor/extensor were evaluated.

Grip force was measured using a Jamar Plus digital hand dynamometer (Sammons Preston, Bolingbrook, IL, USA). Patients were encouraged to exert their maximum grip force in a seated position, with the elbow flexed at 90°. For patients with C5–C7 injuries, the examiner helped them stabilize the wrist with slight extension during testing to prevent the confounding factor of insufficient hand muscle length. Both hands were tested three times, with a 30-s resting interval between each trial. After the first evaluation, rehabilitation programs involving ROM exercise training, strengthening exercises, grip power training (e.g., compressing a softball or a hand grip at least 100 times per day), and re-education of movement strategy training (wrist flexion combined with elbow flexion) were provided. Patients would receive a visit by the orthopedic doctor and therapist at the outpatient clinic after 3 months to ensure that the rehabilitation programs were appropriate. The second evaluation would take place after 6 months.

All data were entered into Excel for further reduction. Continuous data were presented as means and standard deviations, whereas categorical data were described as numbers and proportions. Paired *t*-tests and the sign ranked test were used to determine differences between the first and second evaluations, whereas *t*-tests and chi-square tests were used to compare differences between the C5–C6 and C5–C7 groups. The required sample size was calculated using G*Power version 3.1 software with an alpha of 0.05, and power of 0.8 [19,20]. The minimum required sample was 24 for a *t*-test. Accordingly, 26 subjects were recruited for this study. All statistical analyses were performed using SPSS 24.0, with *p* < 0.05 indicating statistical significance.

## 3. Results

A total of 26 patients (male/female = 22/4) recruited at the initial stage completed the rehabilitation training programs and underwent the second evaluation. The patients had a mean age, weight, height, and BMI of 34.1 ± 10.2 years, 71.5 ± 13.5 kg, 170.4 ± 8.9 cm, and 24.5 ± 3.5 kg/m^2^, respectively. Table 1 shows the characteristics of the patients according to injury levels. No significant differences in age (*p* = 0.062), body weight (*p* = 0.703), body height (*p* = 0.940), or BMI (*p* = 0.540) were observed between C5–C6 and C5–C7 patients.

### 3.1. Outcomes of Functional Performance

**Figure 1:**
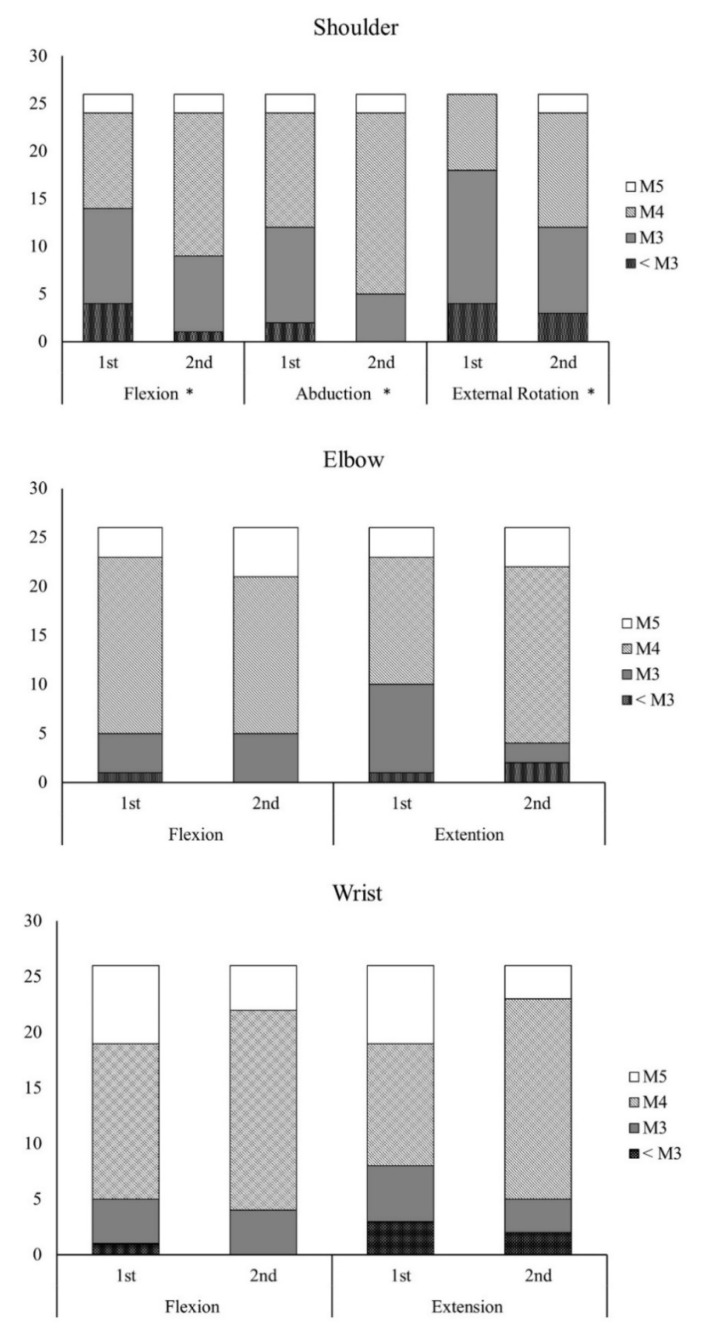
British Medical Research Council scores for shoulder, elbow, and wrist joints during the first and second evaluations (* significant difference between the first and second evaluations, *p* < 0.05).

Table 2 displays the surgical and rehabilitation outcomes of these patients. A follow-up after 6 months showed that patients exhibited improvements in ROM, muscle strength, and grip force. Regarding ROM, a significant difference in elbow flexion was observed between the two evaluations (*p* < 0.001) such that patients could achieve near 130° of flexion after the follow-up. Significant improvements in muscle strength were found in the shoulder flexor (*p* < 0.001), shoulder abductor (*p* < 0.001), shoulder external rotator (*p* = 0.004), elbow flexor (*p* = 0.001), elbow extensor (*p* = 0.034), and grip strength (*p* < 0.001) of the affected arm. Figure 1 shows the BMRC scores in the shoulder, elbow, and wrist joints. Regarding the outcomes of shoulder function, 70% of the patients had scores ≥ M3 at the initial evaluation, whereas more than 80% of patients achieved M3 after 6 months. Significant differences were found in shoulder flexion (*p* = 0.046), shoulder abduction (*p* = 0.013), and shoulder external rotation (*p* = 0.020). Regarding elbow function, more than 80% of the patients achieved a score of M4 in elbow flexion and elbow extension. However, no significant difference in the distribution of BMRC scores was found for the elbow and wrist joints.

### 3.2. Comparisons of Outcomes in Patients with C5–C6 and C5–C7 Injuries

**Figure 2:**
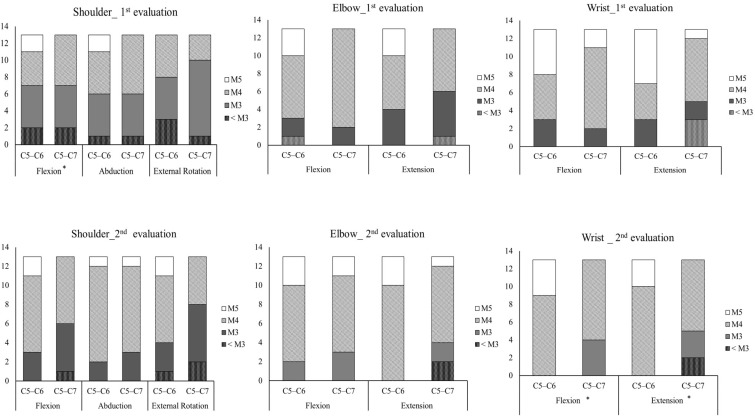
Comparison of British Medical Research Council scores between C5–C6 and C5–C7 BPI patients during the initial and follow-up evaluations (* significant difference between the C5–C6 and C5–C7 BPI groups, *p* < 0.05).

Table 3 reports the outcomes for ROM and muscle strength in C5–C6 and C5–C7 patients. During the first assessment, C5–C6 BPI patients tended to have larger shoulder, elbow, and wrist joint ROM than C5–C7 patients. Significant differences were found in wrist extension (*p* = 0.026). During the second assessment, C5–C6 patients exhibited larger shoulder flexion ROM than C5–C7 patients (*p* = 0.022). A comparison of muscle and grip force in the affected side showed that C5–C6 patients had larger shoulder flexor (*p* = 0.032) and wrists extensor (*p* = 0.017) strength than C5–C7 BPI patients during the first assessment. Meanwhile, significant differences were found in shoulder external rotator (*p* = 0.004), elbow flexor (*p* = 0.028), elbow extensor (*p* = 0.041), wrist extensor (*p* = 0.001), and grip force (*p* = 0.045) of the affected arm during the second assessment. No significant difference in ROM or strength was observed between C5–C6 and C5–C7 BPI patients in the non-affected arm. The Wilcoxon signed-rank tests were used to examine the distributions of BMRC scores between C5–C6 and C5–C7 BPI patients. During the initial evaluation, C5–C6 patients had better BMRC scores for shoulder flexion than C5–C7 patients (*p* = 0.04). During the follow-up evaluation, significant differences in wrist flexion (*p* = 0.018) and wrist extension (*p* = 0.042) were found (Figure 2).

## 4. Discussion

Most of the patients included in our study were able to achieve a BMRC score ≥ M3 in shoulder and elbow function, with differences in quantitative muscle strength improvement between C5–C6 and C5–C7 BPI patients.

Treatment for patients with BPI often set the recovery of shoulder abduction and elbow flexion as the prior goals. Nerve transfer has been the treatment of choice for improving shoulder and elbow function [21,22,23,24,25,26,27,28,29,30]. The number of motor fibers and an appropriate ratio between donor and recipient nerves are critical for a successful nerve transfer reconstruction. Given the large number of motor fibers in the SAN, transferring them to the SSN would be appropriate for optimal shoulder function recovery [6,31]. A meta-analysis also indicated that patients who underwent nerve transfer from SAN to SSN exhibited a better recovery in shoulder abduction [32]. Studies on elbow flexion have shown that an appropriate ratio between the ulnar nerve fascicles and musculocutaneous nerve can support better outcomes after a nerve transfer [33]. Among patients with C5–C6 or C5–C7 lesions, better outcomes were reported after the Oberlin procedure. Bertelli and Ghizoni reported that patients with C5–C6 or C5–C7 lesions had scores > M3 in elbow flexion and extension after a 2-year follow-up [12]. Another study also found that 80% of patients could achieve M3 and showed good function in the biceps 6 months after surgery [14].

High-speed motor vehicle accidents and sports activities are the main causes of traumatic BPIs, with young males showing a higher incidence than females [5]. Ali et al. [30] reviewed the effectiveness of repair techniques in upper BPIs and reported that outcomes did not vary significantly with age, gender distribution, or duration of follow-up. In our study, the ratio of females to males was 2:11 in C5–C6 and C5–C7 BPIs patients. As this ratio is consistent with the epidemiology of BPIs, the results may not be affected by the uneven gender distribution.

Most of our subjects achieved M3 in shoulder and elbow function during the second assessment, with significant differences in BMRC scores having been observed between the first and second assessments. Given the large difference in muscle strength between M3 and M4, as well as the significantly increased values in quantitative strength determined using the handheld dynamometer, the BMRC scoring system may have underestimated improvements in elbow function. Considering the movement characteristics of the elbow joint, elbow strength can be quantitatively evaluated using the lifting test or a customized device. According to definitions established by Waikakul et al., the ability to lift a 2 kg weight more than 30 times was considered an excellent result, whereas the achievement of a BMRC score of ≥M3 despite not having completed the lifting task was considered a good result [29]. After recording the heaviest weights lifted by patients with scores > M3, Teboul et al. reported a mean lifting weight of 4.2 kg [34]. Carlsen et al., who quantitatively assessed elbow flexor strength using a torque sensor, reported that the elbow flexor torque ranged from 2.5% to 56%, contralaterally [1]. The mean isometric strength of the elbow flexor during the first evaluation in our study was 5.9 kg, with our patients showing an improvement by the second evaluation, with a mean strength of 8.0 kg. Additionally, better shoulder function can provide stability during arm movements, which can also be beneficial for elbow and hand movements.

In our study, C5–C7 BPIs patients did not differ significantly from C5–C6 BPIs patients in terms of shoulder abduction, which is one of the goals of reconstruction. However, they showed less improvement in elbow extension, wrist, and grip force, which might be one reason patients are usually unsatisfied with the outcomes after nerve transfer. C5–C7 BPIs patients suffered more severe injuries that needed longer recovery periods. In addition to nerve transfer for shoulder and elbow function, secondary reconstruction for wrist and hand function might be needed to achieve better functional outcomes [2].

In patients undergoing nerve transfer approaches, the morbidity of the donor nerve should be observed, which could be a complication after reconstruction. Previous studies had reported that the Oberlin procedure had little effect on hand function [8,10,34,35] evaluated using grip strength, pinch strength, and two-point discrimination in small fingers before and after surgery in patients who had undergone triple nerve transfer [11]. Their findings showed no functional loss in the hand and no clinical donor nerve deficits. Teboul et al. reported a mean postoperative grip strength of 25.3 kg in patients with C5–C6 and C5–C7 BPI. After comparing the preoperative grip strength, the same study found a mean improvement of 9 kg [34]. In the present study, the mean grip strength during the first evaluation was 21.4 kg, which significantly increased to 24.4 kg during the second evaluation after 6 months of strength training. Although preoperative grip strength was not measured, the mean strength at the last evaluation was similar to that reported in previous studies.

High motivation for intensive postoperative rehabilitation training is an important indication of nerve transfer, and the specific rehabilitation program depends on the donor nerves [16,36]. Chalidapong et al., who compared the bicep’s activation levels during trunk flexion, attempted elbow flexion, forced inspiration, and forced expiration in patients who had received intercostal nerve transfer [37], indicated that elbow flexion combined with trunk flexion induced the greatest activation, suggesting that trunk flexion could be emphasized in the rehabilitation protocol for these patients. In addition, several studies have investigated the concept of donor activation-focused rehabilitation approach (DAFRA). The main focus of DAFRA is to strengthen the altered neural pathway after nerve transfer to optimize the outcome [38]. Patients are required to contract the muscles innervated from the donor nerve to initiate contraction of the reinnervated muscles; otherwise, the patients may adopt compensation strategies that do not optimize the metabolic effort.

Patients who underwent nerve transfer from the ulnar nerve to the musculocutaneous nerve were commonly prescribed grip strengthening in their home exercise program. Several factors can influence postoperative training effects, and spontaneous recovery during the training period cannot be overlooked. In the current study, a comparison of the outcomes between the first and second assessments showed differences in the improvements of C5–C6 and C5–C7 BPI patients. This could help establish reference values for setting rehabilitation goals in BPI patients with various injury levels or for a better understanding of the effects of additional rehabilitation interventions. In addition, quantitative assessments could also help evaluate improvements in functional outcomes of BPI patients after nerve transfer [39]. Another important aspect of rehabilitation is the observation of task performance in isolation, without the initial contraction of the muscles innervated by the donor nerve. To date, little information has been available on the effects of postoperative training in patients with BPI, suggesting the need for future studies on this topic.

There are several possible limitations of our study. One is the relatively small sample size, although we recruited more than the minimum required number of patients according to the power analysis. We recruited upper-type BPIs patients that received a transfer of the spinal accessory nerve to the suprascapular nerve and Oberlin’s method; however, there are various reconstruction approaches or secondary reconstruction methods for BPIs. The assessments used in our study could be applied for outcome evaluation of BPIs, and recruiting more types of BPIs patients could shed light on the recovery progression of various reconstruction methods. As most patients suffered from traffic accidents, it was difficult to obtain performance data before the surgery. Thus, we could not compare pre-operation and post-operation functions.

Furthermore, clinician grading of range of motion and muscle strength may not be sufficient to reflect a patient’s functional level in daily activities. Patient-centered outcome measures, such as the Short Form 36 (SF-36) Disabilities of the Arm, Shoulder and Hand (DASH), VAS pain score, and questionnaires about satisfaction or psychosocial well-being, have been used for outcome evaluation in BPIs [40,41]. Subjective assessment could be taken into consideration, and the correlation with quantitative data can be determined to provide multiple perspectives on the surgical outcome.

## 5. Conclusions

The current study found that transferring the fascicle of the ulnar nerve to the musculocutaneous nerve was an effective approach for restoring elbow flexion function, with no impairment found in the donor nerve upon a follow-up evaluation. Quantitative muscle strength measured using a dynamometer could help surgeons and therapists detect changes accurately. These findings should assist in the establishment of an effective postoperative rehabilitation protocol in patients with BPI.

## Figures and Tables

**Table 1:** Characteristics of subjects with C5–C6 (*n* = 13) and C5–C7 (*n* = 13) injuries.

	C5–C6	C5–C7
Sex (male/female)	11/2	11/2
Age (y/o)	37.9 ± 11.2	30.4 ± 7.9
Body weight (kg)	70.5 ± 15.0	72.5 ± 12.4
Body height (cm)	170.5 ± 9.5	170.3 ± 8.6
BMI (kg/m^2^)	24.1 ± 3.7	24.9 ± 3.3
Injury side (Left/Right)	9/4	5/8
Injury type (number)		
Traffic accident	10	9
Cut injury	1	2
Falling	0	2
Iatrogenic	2	0

**Table 2:** Outcomes for range of motion, muscle strength, and grip force during the first and second evaluations.

		1st Evaluation	2nd Evaluation	*p* Value
Range of motion (degree)				
Shoulder flexion		77.7 ± 47.2	92.2 ± 52.7	0.099
Shoulder abduction		92.13 ± 53.2	127.5 ± 11.3	0.106
Elbow flexion		120.4 ± 14.2	129.5 ± 10.5	<0.001 *
Wrist extension		49.8 ± 11.3	49.4 ± 15.3	0.933
Muscle strength (kgf)				
Shoulder flexor	Affected	5.7 ± 3.1	8.0 ± 3.9	<0.001 *
	Non-affected	20.0 ± 5.8	21.1 ± 5.1	0.159
Shoulder abductor	Affected	6.5 ± 3.8	9.0 ± 4.0	<0.001 *
	Non-affected	17.6 ± 5.6	18.6 ± 4.7	0.155
Shoulder external rotator	Affected	2.8 ± 2.0	3.6 ± 2.2	0.004 *
	Non-affected	13.7 ± 3.8	14.3 ± 3.7	0.235
Elbow flexor	Affected	5.9 ± 4.1	8.0 ± 4.7	0.001 *
	Non-affected	21.5 ± 6.5	21.1 ± 5.2	0.648
Elbow extensor	Affected	6.3 ± 4.5	7.3 ± 4.0	0.034 *
	Non-affected	14.7 ± 4.0	14.4 ± 4.3	0.634
Wrist extensor	Affected	3.9 ± 1.9	4.7 ± 2.2	0.007 *
	Non-affected	9.3 ±3.2	10.0 ±2.7	0.153
Wrist flexor	Affected	5.1 ± 2.5	6.1 ± 2.5	0.338
	Non-affected	11.3 ± 3.4	13.6 ± 4.2	0.003 *
Grip strength	Affected	21.4 ± 11.3	24.4 ± 12.0	<0.001 *
	Non-affected	39.9 ± 11.7	41.6 ± 13.1	0.065

* Significant difference between the first and second evaluations, *p* < 0.05.

**Table 3:** Comparisons of range of motion, muscle strength, and grip force between patients with C5–C6 and C5–C7 brachial plexus injuries.

Functional Outcomes		C5–C6	C5–C7	*p* Value
Range of motion (degree)				
Shoulder flexion	1st	96.2 ± 54.6	50.0 ± 4.1	0.137
	2nd	121.2 ± 46.6	48.8 ± 22.9	0.022 *
Shoulder abduction	1st	133.3 ± 10.4	124.0 ± 11.2	0.293
	2nd	110.8 ± 51.8	51.8 ± 12.9	0.060
Elbow flexion	1st	122.9 ± 12.5	118.1 ± 15.8	0.406
	2nd	131.4 ± 11.3	128.5 ± 9.8	0.488
Wrist extension	1st	55.5 ± 9.0	38.3 ± 7.6	0.026 *
	2nd	53.8 ± 16.8	40.7 ± 7.5	0.248
Muscle Strength (Affected arm, kgf)				
Shoulder flexor	1st	6.8 ± 3.3	4.2 ± 2.1	0.032 *
	2nd	9.3 ± 3.7	6.5 ± 3.7	0.071
Shoulder abductor	1st	7.1 ± 2.9	5.9 ± 4.6	0.445
	2nd	9.8 ± 2.8	8.3 ± 5.0	0.366
Shoulder external rotator	1st	3.3 ± 2.5	2.3 ± 1.2	0.212
	2nd	4.8 ± 2.4	2.5 ± 1.2	0.004 *
Elbow flexor	1st	7.3 ± 4.1	4.6 ± 3.7	0.090
	2nd	10.0 ± 4.0	6.0 ± 4.7	0.028 *
Elbow extensor	1st	7.7 ± 3.7	4.6 ± 4.7	0.083
	2nd	8.6 ± 3.4	5.4 ± 4.2	0.041 *
Wrist extensor	1st	4.7 ± 1.8	3.0 ± 1.6	0.017 *
	2nd	6.0 ± 2.1	3.1 ± 1.5	0.001 *
Wrist flexor	1st	5.1 ± 3.2	5.2 ± 1.2	0.924
	2nd	7.4 ± 2.2	4.3 ± 1.8	0.051
Grip force	1st	25.4 ± 12.8	17.4 ± 8.3	0.070
	2nd	29.1 ± 12.9	19.7 ± 9.3	0.045 *

* Significant difference between C5–C6 and C5–C7 groups, *p* < 0.05.
